# The Long-Term Cardiovascular Impact of COVID-19: Pathophysiology, Clinical Manifestations, and Management

**DOI:** 10.7759/cureus.66554

**Published:** 2024-08-10

**Authors:** Rushi V Mukkawar, Harshitha Reddy, Nishant Rathod, Sunil Kumar, Sourya Acharya

**Affiliations:** 1 Internal Medicine, Jawaharlal Nehru Medical College, Datta Meghe Institute of Higher Education and Research, Wardha, IND

**Keywords:** post-acute sequelae of covid-19 (pasc), endothelial dysfunction, myocarditis, sars-cov-2, cardiovascular complications, long covid

## Abstract

The COVID-19 pandemic, caused by the novel coronavirus SARS-CoV-2, has resulted in a substantial global health crisis, with effects extending far beyond the acute phase of infection. This review aims to provide a comprehensive overview of the long-term cardiovascular impact of COVID-19, focusing on the pathophysiology, clinical manifestations, diagnostic approaches, management strategies, and future research directions. SARS-CoV-2 induces cardiovascular complications through mechanisms such as inflammation, endothelial dysfunction, and direct myocardial injury, leading to conditions like myocarditis, heart failure, arrhythmias, and thromboembolic events. These long-term effects, collectively called "long COVID" or post-acute sequelae of SARS-CoV-2 infection (PASC), present significant challenges for healthcare systems and patient management. Diagnostic approaches include imaging techniques and laboratory tests to identify and monitor cardiovascular complications. Management strategies emphasize a holistic approach, incorporating pharmacological treatments and lifestyle modifications. Special attention is required for vulnerable populations, including those with pre-existing cardiovascular conditions. Ongoing research is essential to understand the full spectrum of long-term cardiovascular impacts and to develop effective treatments. This review highlights the critical need for continued vigilance, multidisciplinary care, and research to address the cardiovascular sequelae of COVID-19 and improve long-term health outcomes for survivors.

## Introduction and background

COVID-19, caused by the novel coronavirus SARS-CoV-2, was first identified in December 2019 in Wuhan, China, and rapidly escalated into a global pandemic [1]. The virus primarily spreads through respiratory droplets and has many clinical manifestations, from asymptomatic infection to severe respiratory illness and death [2]. The pandemic has profoundly impacted global health, economies, and daily life. As of mid-2024, COVID-19 has caused millions of deaths worldwide and has led to significant healthcare challenges [3]. The initial focus of COVID-19 research and treatment was on the acute phase of the disease, addressing symptoms such as fever, cough, and severe respiratory distress. However, as the pandemic has progressed, it has become evident that a substantial number of individuals experience persistent symptoms and health issues long after the acute phase of the infection has resolved. These long-term effects, collectively referred to as "Long COVID" or post-acute sequelae of SARS-CoV-2 infection (PASC), affect multiple organ systems, including the cardiovascular system [4].

Understanding the long-term effects of COVID-19 is crucial for several reasons. Firstly, it helps provide comprehensive care to patients suffering from symptoms long after their initial infection. Secondly, it aids in identifying potential risk factors for developing long-term complications, which can inform public health strategies and guidelines [5]. Thirdly, it highlights the need for ongoing research and resources to manage the healthcare burden posed by Long COVID. The cardiovascular system is particularly affected in many COVID patients. Complications, such as myocarditis, heart failure, arrhythmias, and thromboembolic events, have been reported. These conditions not only affect the quality of life of survivors but also pose significant long-term health risks. Thus, a detailed understanding of the pathophysiology, clinical manifestations, and management strategies for the cardiovascular impact of COVID-19 is essential [6].

This review aims to provide a comprehensive overview of the long-term cardiovascular impact of COVID-19. It aims to elucidate the pathophysiological mechanisms through which COVID-19 affects the cardiovascular system. Additionally, the review will describe the clinical manifestations and symptoms related to long-term cardiovascular involvement and discuss the diagnostic approaches and tools used to identify cardiovascular complications in COVID-19 survivors. Furthermore, it will review the current management strategies and treatments available for addressing these complications, highlight the prognosis and long-term outcomes for patients with cardiovascular involvement post-COVID-19, identify special populations at risk, and discuss considerations for these groups. Finally, the review will summarize the ongoing research and future directions needed to better understand and manage the long-term cardiovascular impact of COVID-19. This review aims to serve as a valuable resource for healthcare providers, researchers, and policymakers involved in the care and study of COVID-19 survivors, ensuring that they are equipped with the latest knowledge and insights to tackle the long-term cardiovascular consequences of the pandemic.

## Review

Pathophysiology of COVID-19 and cardiovascular system

Mechanisms of SARS-CoV-2 Infection

SARS-CoV-2, the virus responsible for COVID-19, infects host cells through a complex, multistep process involving its spike (S) protein and specific cellular receptors. The process begins with binding the S protein to the angiotensin-converting enzyme 2 (ACE2) receptor, primarily located on the surface of various human cells, including those in the respiratory tract [7]. The receptor-binding domain (RBD) of the S1 subunit of the S protein mediates this interaction. Additionally, SARS-CoV-2 can utilize alternative receptors, such as neuropilin-1 (NRP1) and CD147, potentially enhancing its ability to infect different tissues compared to its predecessor, SARS-CoV [8]. Following receptor binding, the S1 subunit of the S protein is shed, revealing the S2 subunit. The S2 subunit is critical for the fusion of the viral membrane with the host cell membrane. This fusion process is facilitated by the proteolytic cleavage of the S protein by host proteases, including furin, transmembrane protease serine 2 (TMPRSS2), and cathepsins [9]. This cleavage is essential for effective viral entry into the host cell. Once the viral and host membranes fuse, the viral genome is released into the host cell's cytoplasm, allowing the virus to exploit the host's cellular machinery to replicate and assemble new viral particles [10]. During close contact between infected and uninfected individuals, SARS-CoV-2 primarily spreads through respiratory droplets and fomites. The virus can spread rapidly, particularly in crowded or poorly ventilated settings [11]. Infection with SARS-CoV-2 can result in a range of symptoms, from mild respiratory illness to severe conditions such as acute respiratory distress syndrome (ARDS) and multi-organ failure. The pathogenesis of COVID-19 is complex, involving direct viral effects and an exaggerated immune response that can contribute to tissue damage and systemic complications [11].

Impact on the Cardiovascular System

COVID-19 has significant and enduring impacts on the cardiovascular system, affecting both individuals with pre-existing conditions and those who were previously healthy. One of the primary mechanisms by which the virus affects the heart is through direct viral infection and inflammation. SARS-CoV-2, the virus responsible for COVID-19, enters host cells by binding to the ACE2 receptor, which is highly expressed in cardiovascular tissues [12]. This interaction can lead to myocarditis, an inflammation of the heart muscle, and acute cardiac injury. The inflammatory response, often marked by a cytokine storm, can further damage the heart and blood vessels, leading to severe cardiovascular complications [13]. Beyond direct injury, COVID-19 induces a hypercoagulable state, significantly increasing the risk of thromboembolic events. Patients with COVID-19 are more susceptible to conditions such as venous thromboembolism (VTE) and pulmonary embolism (PE). The inflammatory response associated with the virus can promote the formation of neutrophil extracellular traps (NETs), contributing to clot formation and vascular occlusion. These thrombotic events can severely compromise cardiovascular function, leading to acute coronary syndromes and other serious complications [14]. Arrhythmias and heart failure are also prevalent cardiovascular manifestations of COVID-19. Many patients experience various types of arrhythmias, including atrial fibrillation and ventricular tachycardia, often related to the stress imposed on the heart by the infection. Furthermore, COVID-19 can lead to left ventricular dysfunction and acute right ventricular failure, raising the risk of heart failure. The interplay between the viral infection and the heart's response can result in long-term cardiac complications, even in patients initially presenting with mild symptoms [15]. Individuals with pre-existing cardiovascular conditions, such as hypertension, coronary artery disease, and diabetes, face an even greater risk of severe outcomes from COVID-19. The infection can exacerbate these underlying conditions, leading to poorer prognoses and increased mortality rates. This underscores the bidirectional relationship between COVID-19 and cardiovascular health, as existing heart conditions can complicate the course of the disease, while COVID-19 can further impair cardiovascular function [16].

Inflammatory Response and Cytokine Storm

The inflammatory response and cytokine storm associated with COVID-19 are critical factors influencing disease severity and patient outcomes. The inflammatory response is a natural defense mechanism activated when the body encounters pathogens like SARS-CoV-2. However, in COVID-19, this response can become dysregulated, leading to a hyperinflammatory state [17]. Upon infection, the innate immune system acts as the initial line of defense, activating immune cells that release cytokines and interferons to initiate the adaptive immune response. This response can escalate uncontrollably in severe cases, resulting in excessive inflammation. Cytokines are signaling proteins that mediate and regulate immunity and inflammation. In COVID-19, key cytokines, such as IL-6, IL-10, TNF-α, and chemokines like CCL2 and CXCL8 are significantly elevated. These cytokines contribute to an inflammatory cascade that can damage tissue and multi-organ failure [18]. A cytokine storm is an overproduction of cytokines, leading to severe systemic inflammation. This phenomenon is particularly concerning in COVID-19 patients and is associated with worse clinical outcomes. The immune response to SARS-CoV-2 primarily drives the cytokine storm in COVID-19. The virus's S protein and various immune cells, including macrophages and T cells, play a significant role in this hyperinflammatory response. The storm can cause widespread tissue damage, particularly in the lungs, leading to ARDS and other complications [19]. The elevation of pro-inflammatory cytokines correlates with disease severity and mortality. Patients experiencing a cytokine storm may develop complications, such as ARDS, cardiac injury, and coagulopathy, which can further exacerbate their condition. Even after recovery from acute COVID-19, elevated cytokine levels can persist, potentially contributing to post-COVID conditions and long-term health issues. This prolonged inflammation may lead to chronic symptoms and complications, necessitating ongoing monitoring and management [20]. Given the detrimental effects of the inflammatory response and cytokine storm in COVID-19, various therapeutic strategies are being explored. Medications, such as corticosteroids (e.g., dexamethasone), have reduced mortality in severe COVID-19 cases by suppressing the inflammatory response. Targeting specific cytokines involved in the storm, such as IL-6 inhibitors (e.g., tocilizumab), is being investigated to mitigate the hyperinflammatory response and improve patient outcomes. Additionally, vaccines may help modulate the immune response, potentially reducing the risk of severe inflammation and cytokine storms in future infections [21].

Endothelial Dysfunction and Thrombosis

Endothelial dysfunction and thrombosis are interrelated phenomena that significantly impact various cardiovascular diseases. Understanding their relationship is essential for developing effective therapeutic strategies. Endothelial dysfunction refers to impaired endothelium functioning, the thin layer of cells lining blood vessels. This dysfunction can be triggered by inflammation, oxidative stress, and abnormal blood flow patterns. Conditions like hypertension, diabetes, and hyperlipidemia can cause chronic inflammation, damaging endothelial cells and disrupting their normal function [22]. An imbalance between the production of reactive oxygen species (ROS) and the body's ability to neutralize them leads to oxidative damage of endothelial cells, impairing their capacity to regulate vascular tone and maintain homeostasis. Additionally, abnormal blood flow patterns can induce changes in endothelial cell function, contributing to atherogenesis and thrombosis. The consequences of endothelial dysfunction include reduced nitric oxide (NO) availability, increased vascular permeability, and heightened expression of adhesion molecules, facilitating leukocyte adhesion and migration and setting the stage for atherogenesis and thrombosis [22]. Thrombosis, the formation of a blood clot within a blood vessel, can obstruct blood flow and occur in either arteries (arterial thrombosis) or veins (venous thrombosis), each with distinct mechanisms and outcomes. Arterial thrombosis is often linked to atherosclerosis, where the rupture of an atherosclerotic plaque triggers platelet activation and coagulation cascade initiation, potentially leading to myocardial infarction or stroke. Deep vein thrombosis (DVT) is a common type of venous thrombosis, often precipitated by factors such as prolonged immobility, surgery, or malignancy. Virchow's triad-blood flow stasis, endothelial injury, and hypercoagulability describe the conditions that promote venous thrombus formation [23]. Endothelial dysfunction plays a critical role in thrombosis development. Dysfunctional endothelium fosters a pro-thrombotic environment by increasing the expression of tissue factors and reducing the availability of anticoagulant factors like thrombomodulin. Endothelial injury exposes subendothelial collagen and von Willebrand factor, leading to platelet adhesion and activation-a key step in thrombus formation [24]. Inflammatory cytokines released during endothelial dysfunction can enhance the coagulation cascade, further promoting thrombosis. Elevated levels of IL-6 and TNF-α, for example, can increase the expression of pro-coagulant factors. Chronic endothelial dysfunction can also induce structural changes in blood vessels, such as increased stiffness and reduced luminal diameter, contributing to elevated shear stress and further endothelial injury, creating a vicious cycle [25].

Myocardial Injury and Myocarditis

Myocardial injury and myocarditis are notable complications of COVID-19, impacting patient outcomes and long-term cardiovascular health. Understanding their mechanisms, clinical manifestations, and implications is essential for effective management. Myocardial injury in the context of COVID-19 is characterized by elevated levels of cardiac troponin, a biomarker indicating damage to the heart muscle. Studies show that approximately 25% of COVID-19 patients experience myocardial injury, which can occur even in those without pre-existing cardiovascular conditions [26]. This injury may arise from several mechanisms: direct viral effects, where SARS-CoV-2 infects cardiac cells, causing cellular damage and inflammation; systemic inflammation, triggered by the virus and often resulting in a cytokine storm that can damage myocardial tissue and disrupt cardiac function; endothelial dysfunction, where COVID-19 causes endothelial cell activation and microvascular thrombosis, contributing to myocardial ischemia and injury; and increased cardiac demand, where respiratory distress associated with COVID-19 places additional strain on the heart, exacerbating myocardial injury. Patients with myocardial injury related to COVID-19 often face higher mortality rates and may develop long-term complications such as heart failure and arrhythmias. Diagnosing myocardial injury can be challenging due to atypical presentations and symptom overlap with severe COVID-19 pneumonia [27]. Myocarditis, an inflammation of the heart muscle, can occur as a direct consequence of viral infection or as a secondary effect of the immune response. It is recognized as a complication in COVID-19 patients, particularly those with severe disease or pre-existing cardiovascular conditions. The risk is notably higher in younger individuals and athletes, leading to increased scrutiny in these populations [28]. Symptoms of myocarditis may include chest pain, shortness of breath, and palpitations, though many cases may be asymptomatic or present with atypical symptoms, complicating diagnosis. Diagnosis typically involves imaging studies such as echocardiography, cardiac MRI, and biomarker assessment. Management may include supportive care, anti-inflammatory medications, and monitoring for complications such as heart failure or arrhythmias. Patients recovering from myocarditis may face long-term cardiovascular risks, including chronic heart failure and reduced exercise tolerance, necessitating ongoing cardiovascular evaluation [29].

Clinical manifestations of long-term cardiovascular impact

Acute vs. Long-Term Cardiovascular Symptoms

During the acute phase of COVID-19, patients may experience a range of cardiovascular symptoms that can signal severe disease and potential complications. One of the most critical indicators is myocardial injury, often assessed through elevated cardiac troponin levels. This biomarker is associated with increased illness severity and higher mortality rates [30]. COVID-19 can also lead to myopericarditis, which involves inflammation of the heart muscle (myocarditis) and the surrounding pericardial tissue (pericarditis). Another acute manifestation is stress cardiomyopathy, also known as Takotsubo syndrome, characterized by a temporary and reversible weakening of the heart muscle, typically triggered by emotional or physical stress. Arrhythmias, or abnormal heart rhythms, are prevalent during the acute phase, with conditions such as atrial fibrillation frequently observed. Additionally, COVID-19 significantly increases the risk of thromboembolic events, including strokes and myocardial infarctions, due to the inflammatory response and endothelial dysfunction caused by the virus [31]. In the aftermath of COVID-19, many survivors report long-term cardiovascular symptoms that can persist for months or even years. Chest pain is among the most common complaints, affecting up to 20% of patients around 60 days post-infection. Palpitations, or the sensation of a racing or irregular heartbeat, are also frequently reported, with studies indicating that approximately 9% of patients experience this symptom six months after their acute illness [32]. Additionally, some individuals may develop dysautonomia, a condition characterized by autonomic nervous system dysfunction that can lead to symptoms such as palpitations and dizziness. Myocarditis can have long-term implications, potentially evolving into overt or subclinical myocardial dysfunction that affects overall heart health. The risk of arrhythmias remains elevated in COVID-19 survivors, contributing to ongoing cardiovascular concerns. Furthermore, there is a significant increase in the risk of heart failure among those who have recovered from COVID-19, even among individuals who were not hospitalized during the acute phase of the illness [33].

PASC or Long COVID

PASC, commonly known as long-term COVID, involves a range of symptoms that persist for weeks or months after the acute phase of SARS-CoV-2 infection. This condition has become increasingly recognized as a significant public health issue, affecting a substantial number of individuals who have recovered from COVID-19 [34]. The National Institutes of Health (NIH) defines PASC as symptoms lasting at least four weeks post-infection that cannot be attributed to other diagnoses. Research suggests that approximately 54% to 80% of COVID-19 survivors report experiencing at least one long-term symptom, with about 10% of patients developing persistent symptoms lasting several months [35]. The clinical manifestations of PASC can vary widely among individuals. Commonly reported symptoms include fatigue, respiratory, neurological, cardiovascular, psychiatric, and gastrointestinal issues. Fatigue is a prevalent complaint, often debilitating and significantly impacting daily activities. Persistent cough, dyspnea (shortness of breath), and chest tightness are frequently observed, especially among those who have severe COVID-19 [36]. Cognitive difficulties, often referred to as "brain fog," along with headaches, dizziness, and concentration issues, are also common. Cardiovascular symptoms, such as palpitations and chest pain, may be present, and individuals might face an increased risk of cardiovascular diseases, even if they were previously healthy. Mental health issues, such as anxiety and depression, further complicate recovery, and some individuals report ongoing gastrointestinal problems, including nausea and changes in appetite [37]. The underlying mechanisms of PASC are not yet fully understood, but several hypotheses exist, including immune dysregulation, organ damage, viral reservoirs, and microbiome interactions [38]. Persistent inflammation and immune system activation may contribute to ongoing symptoms, potentially leading to autoimmune responses. Damage to various organ systems during the acute phase of COVID-19, including the lungs, heart, and brain, may result in long-term effects. There is speculation that remnants of the virus may persist in certain tissues, causing ongoing symptoms. Additionally, changes in the microbiome due to COVID-19 might influence recovery and symptom persistence [38]. Managing PASC requires a multidisciplinary approach focused on symptom relief and rehabilitation. Key strategies include regular follow-ups to effectively assess and manage symptoms, tailored rehabilitation programs, such as physical therapy and cognitive rehabilitation, to restore function and improve quality of life, addressing mental health issues through counseling and psychiatric support, and ongoing research to better understand PASC and develop effective treatments. Initiatives like the NIH's Researching COVID to Enhance Recovery (RECOVER) study aim to gather data on the long-term effects of COVID-19 and identify potential therapeutic targets [39].

Common Cardiovascular Symptoms in Long COVID

**Figure 1:**
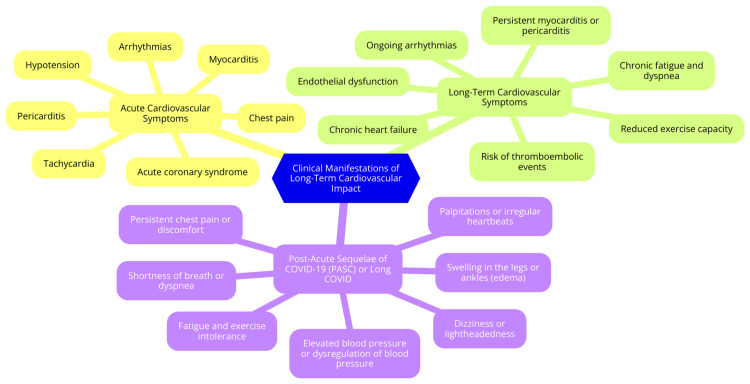
Clinical manifestations of long-term cardiovascular impact Image credit: Dr. Rushi V. Mukkawar

Long COVID has been associated with a range of persistent cardiovascular symptoms that can significantly impact the quality of life of individuals who have recovered from the acute phase of COVID-19. Among the most common symptoms are fatigue and palpitations. Persistent fatigue is often described as an overwhelming tiredness that does not improve with rest, leaving individuals feeling drained and unable to engage in daily activities [34]. This fatigue can be debilitating, affecting both physical and mental well-being. Palpitations, characterized by rapid or irregular heartbeat sensations, are also frequently reported. While palpitations may be linked to anxiety and stress, in the context of long-term COVID, they may suggest underlying cardiac issues that require further investigation [34]. In addition to fatigue and palpitations, many individuals experience chest pain and dyspnea. Chest pain is a prevalent symptom, with studies indicating that a significant percentage of long-term COVID patients report discomfort or pain in the chest area [40]. This pain can vary in intensity and may be accompanied by sensations of tightness, which can be alarming and often lead to concerns about cardiac health. Dyspnea, or shortness of breath, is another common complaint among long COVID survivors. This symptom can occur during physical exertion or even at rest, reflecting potential complications affecting the lungs and the cardiovascular system [41]. These symptoms highlight the complex relationship between COVID-19 and cardiovascular health, underscoring the need for careful monitoring and management of affected individuals. As healthcare providers continue to understand long COVID, recognizing these cardiovascular manifestations will be crucial for developing effective treatment strategies and improving patient outcomes [42]. Clinical manifestations of long-term cardiovascular impact are shown in Figure 1.

Specific Cardiovascular Conditions

The long-term cardiovascular impact of COVID-19 encompasses several significant conditions that have emerged as major concerns for healthcare providers and patients. Among these, myocarditis and pericarditis are notable. Myocarditis, characterized by heart muscle inflammation, and pericarditis, which involves inflammation of the pericardium (the membrane surrounding the heart), can occur simultaneously as myopericarditis. The incidence of these conditions has markedly increased during the pandemic, with estimates suggesting a 15-fold rise compared to pre-COVID levels [15]. Reports indicate incidence rates ranging from 150 to 4,000 cases per 100,000 individuals, particularly affecting young males under 50 years old. Symptoms of myocarditis and pericarditis often include chest pain, shortness of breath, palpitations, fever, cough, and syncope. These symptoms can mimic those of other viral infections, necessitating careful evaluation for accurate diagnosis. The underlying pathophysiology often involves the body’s immune response to the viral infection, leading to cardiac damage, which can be reflected in elevated biomarkers such as troponins and N-terminal pro-B-type natriuretic peptide (NT-proBNP) [43]. Another significant condition linked to COVID-19 is heart failure, which can manifest in both acute and chronic forms. Individuals with pre-existing cardiovascular conditions are particularly vulnerable to developing heart failure post-COVID. The virus may exacerbate underlying heart issues or induce new cardiac dysfunction due to direct myocardial damage or secondary effects from systemic inflammation. Symptoms of heart failure, including fatigue, dyspnea (shortness of breath), and fluid retention, can severely impair quality of life and functional capacity, highlighting the need for ongoing monitoring and management in affected individuals [44]. Arrhythmias are also prevalent among COVID-19 patients, with a notable increase in their incidence. Common arrhythmias observed include atrial fibrillation and ventricular arrhythmias, which may arise from myocardial inflammation, electrolyte imbalances, or autonomic dysfunction caused by viral infection. Patients presenting with palpitations or other arrhythmia-related symptoms require thorough cardiac evaluation, including ECG monitoring, and may need treatment with antiarrhythmic medications to manage their condition effectively [45]. Finally, COVID-19 is associated with an increased risk of thromboembolic events, such as DVT, PE, and strokes. The hypercoagulable state induced by the virus is thought to result from inflammation and endothelial dysfunction. Healthcare providers must monitor patients who have recovered from COVID-19 for signs of thromboembolic complications, especially if they present with sudden onset of chest pain, shortness of breath, or neurological symptoms [46].

Diagnostic approaches

Initial Assessment and History-Taking

Initial assessment and history taking are critical components in managing patients suspected of having COVID-19. This process involves evaluating symptoms, exposure history, and overall clinical status to determine the appropriate course of action [4]. The initial assessment should follow a structured approach for screening and triaging patients. Healthcare providers should inquire about new symptoms such as fever, cough, shortness of breath, muscle aches, sore throat, runny nose, and loss of smell or taste [47]. Exposure history is also essential; questions should include whether the patient has tested positive for COVID-19 in the past 14 days or has been in close contact with someone with COVID-19. Based on these responses, patients are categorized by their likelihood of having COVID-19, which helps prioritize care. Patients with acute or unstable conditions should receive immediate attention, while others may need further evaluation. Classification into categories, such as confirmed cases, suspect cases, and asymptomatic exposed individuals, aids in managing isolation and treatment protocols [47]. During history taking, healthcare providers should focus on clinical, epidemiological, and social history. The clinical history should document any previous medical conditions, current medications, and allergies to assess the patient's overall health status and potential risks [48]. The epidemiological history should include recent travel, contact with confirmed cases, and symptoms experienced before the visit. Understanding the patient's living situation, occupation, and potential exposure risks can provide additional context for their health status [48]. Initial assessment recommendations include using personal protective equipment (PPE). Patients should wear surgical masks upon entering healthcare facilities to minimize the risk of transmission. Those suspected of having COVID-19 should be directed to a separate area to prevent exposure to others. Vital signs should be monitored regularly, and any changes in patient condition should be documented and addressed promptly [49].

Imaging Techniques

Echocardiography is a widely available and cost-effective imaging tool crucial for evaluating cardiac structure and function, especially in patients with COVID-19. This technique provides real-time assessment of the heart, offering valuable insights into cardiac function, chamber sizes, and valve integrity [50]. In acute settings, echocardiography is instrumental in guiding management decisions and monitoring hemodynamic status. It can detect subclinical myocardial dysfunction, even in hospitalized patients without obvious cardiac symptoms. Additionally, echocardiographic changes in right and left ventricular strain have demonstrated prognostic value, aiding clinicians in assessing the severity of cardiac involvement in COVID-19 patients [50]. Cardiac MRI is a more advanced imaging modality that comprehensively evaluates myocardial injury, making it especially valuable for assessing COVID-19 survivors. This technique provides high-resolution images that detect subtle changes in myocardial tissue, such as inflammation, edema, and fibrosis [51]. Studies have shown that cardiac MRI can identify distinct patterns of myocardial injury in patients recovering from COVID-19, particularly those with elevated troponin levels during their illness. Due to its detailed visualization of cardiac structure and function, cardiac MRI is recommended to evaluate individuals with a history of COVID-19, particularly those experiencing persistent symptoms or complications [51]. CT angiography is another important imaging technique, particularly useful for diagnosing acute coronary syndromes and PE in COVID-19 patients. This modality allows for rapid visualization of coronary arteries and can identify blockages or abnormalities related to the virus's inflammatory effects [52]. In COVID-19 patients, CT angiography can also reveal changes such as a D-shaped left ventricle, indicative of right ventricular strain, and increased tricuspid regurgitation jet velocity, often associated with PE. The ability to quickly assess these conditions makes CT angiography a valuable tool for managing cardiovascular complications in COVID-19 patients [52].

Laboratory Tests

Assessing cardiac and inflammatory biomarkers plays a crucial role in managing COVID-19 patients. Elevated levels of cardiac biomarkers, such as troponins and natriuretic peptides, indicate myocardial injury and correlate strongly with disease severity and mortality [53]. Troponin elevation, in particular, is closely associated with myocardial damage and has been linked to increased mortality and adverse outcomes in COVID-19 patients. As such, it is a critical marker for assessing cardiac involvement and guiding treatment decisions. Natriuretic peptides, including NT-proBNP, are indicative of heart failure and provide insights into cardiac stress and dysfunction. Elevated NT-proBNP levels are also associated with poorer outcomes, especially in patients presenting with chest pain [54]. Inflammatory markers are essential for understanding the systemic inflammatory response elicited by COVID-19. C-reactive protein (CRP) is an acute-phase reactant that frequently rises in inflammatory conditions, including COVID-19. High CRP levels can indicate severe disease and correlate with worse outcomes. IL-6, a key mediator of inflammation, has been associated with severe cases of COVID-19. Elevated IL-6 levels may predict the need for intensive care and are potential targets for specific therapies. Ferritin, another important inflammatory marker, can indicate a hyper-inflammatory response linked to severe disease and poor prognosis in COVID-19 patients [55]. Additionally, elevated D-dimer levels, primarily a marker for thrombosis, can indicate an increased risk of thromboembolic events in COVID-19 patients. This further complicates cardiac health and management. Monitoring these biomarkers is essential for optimizing patient management and improving outcomes in those affected by COVID-19 [56].

Role of Stress Testing

Stress testing is crucial in various domains, notably finance and healthcare, for evaluating resilience and performance under challenging conditions. In the financial sector, stress testing is vital to risk management. It helps banks and financial institutions identify vulnerabilities by assessing their financial stability against adverse economic downturns or market shocks [57]. Identifying these vulnerabilities is essential for effective risk management. Additionally, regulatory compliance is key, as regulators have stressed the importance of stress testing for monitoring systemic risk and ensuring institutions maintain adequate capital buffers. Stress tests also support strategic decision-making by helping banks determine their risk appetites and decide which business segments to expand or reduce, which is crucial for long-term sustainability and operational integrity. Effective stress testing involves creating realistic and severe scenarios to evaluate potential impacts on financial stability, including scenarios related to credit defaults, liquidity crises, or operational disruptions [57]. In healthcare, particularly cardiology, stress testing is used to evaluate heart function and diagnose cardiovascular conditions. Exercise stress tests monitor how well the heart responds to increased physical activity, helping to identify issues such as coronary artery disease or arrhythmias [58]. This is achieved by measuring heart rate, blood pressure, and electrical activity during exercise. Stress tests are often performed before surgeries to assess the risk of complications related to heart disease, especially in patients with risk factors such as diabetes or hypertension. Results from stress tests can guide healthcare providers in determining the need for more invasive procedures or adjustments to treatment plans to mitigate the risk of heart attacks [58].

Management strategies

General Management Principles

Effective management of cardiovascular disease, especially in the context of COVID-19, requires a robust approach to monitoring and follow-up. Regular screening for hypertension, diabetes, and hyperlipidemia is crucial for identifying and managing cardiovascular risk factors. The challenges presented by the pandemic have made telemedicine an invaluable tool for remote monitoring and consultations [59]. This approach allows healthcare providers to maintain patient engagement and ensure adherence to treatment plans while reducing the risk of virus transmission associated with in-person visits. Patients are also encouraged to monitor their symptoms closely, including chest pain, shortness of breath, palpitations, and dizziness. Prompt medical care is essential for those experiencing these symptoms, particularly COVID-19 survivors who may face an increased risk of cardiovascular complications. Regular follow-ups with healthcare providers are recommended to ensure ongoing assessment of cardiovascular health and timely interventions when necessary [59]. A multidisciplinary approach is crucial for comprehensively managing patients with cardiovascular diseases during the COVID-19 pandemic. This involves collaboration among cardiologists, primary care providers, and specialists in infectious diseases to deliver coordinated care tailored to individual patient needs. Such collaboration is especially important when considering treatment options, including intervention strategies, escalation to mechanical circulatory support, transplantation, or palliative care [60]. Engaging a multidisciplinary team allows for thoroughly exploring therapeutic goals, such as correcting metabolic disturbances, optimizing cardiac preload and afterload, and restoring organ perfusion. In severe cases, this may require the use of vasoactive drugs and careful consideration of candidacy for advanced interventions. By fostering a collaborative environment, healthcare providers can ensure that patients receive comprehensive, patient-centered care that addresses their cardiovascular and overall health needs during these challenging times [60].

Pharmacological Treatments

Pharmacological treatments for cardiovascular complications associated with COVID-19 involve various classes of medications aimed at managing inflammation, coagulopathy, heart failure, and arrhythmias. Anti-inflammatory agents are crucial for managing systemic inflammation caused by COVID-19. Dexamethasone has been widely used to reduce mortality in severe cases by mitigating the inflammatory response. Other anti-inflammatory medications, such as colchicine, have also been studied for their potential to reduce inflammation and improve outcomes in COVID-19 patients, particularly those with cardiovascular comorbidities [61]. COVID-19 increases the risk of thromboembolic events due to coagulopathy, leading to complications such as VTE. Anticoagulants, particularly low-molecular-weight heparin (LMWH), are recommended for thromboprophylaxis in hospitalized COVID-19 patients. Evidence suggests that anticoagulation therapy may lower mortality rates, particularly in patients with elevated D-dimer levels or those meeting sepsis-induced coagulopathy criteria. However, anticoagulation therapy's optimal dosing and duration remain under investigation, highlighting the need for further research [62]. For patients with pre-existing heart failure or those who develop heart failure as a result of COVID-19, careful management is required. Angiotensin-converting enzyme (ACE) inhibitors and angiotensin II receptor blockers (ARBs) are often continued, as they have demonstrated benefits in heart failure management. These medications may also offer protective effects against the severity of COVID-19, though their use should be monitored due to potential interactions with COVID-19 treatments [63]. Arrhythmias are common in COVID-19 patients, particularly among those with severe disease. Amiodarone, an antiarrhythmic drug, has been suggested for use in this population to prevent sudden cardiac arrest. It has shown in vitro activity against SARS-CoV, indicating a potential dual role in managing arrhythmias and mitigating viral spread. Other antiarrhythmic agents may also be considered based on the patient's clinical presentation and underlying conditions [45].

Non-pharmacological Interventions

Non-pharmacological interventions are essential in managing cardiovascular health, particularly for COVID-19 survivors. These strategies focus on lifestyle modifications, rehabilitation programs, and psychological support, which are crucial in mitigating the long-term cardiovascular risks associated with the virus [64]. Lifestyle modifications are critical for individuals recovering from COVID-19. Regular physical activity is strongly encouraged, as it can significantly improve cardiovascular fitness and overall health. Engaging in activities such as walking, cycling, or structured exercise programs can enhance heart health and lower the risk of cardiovascular complications [64]. A balanced diet of fruits, vegetables, whole grains, and lean proteins is vital for managing blood pressure, cholesterol levels, and body weight. Smoking cessation is another key component of lifestyle modification, as quitting smoking can dramatically reduce the risk of cardiovascular disease and improve overall health outcomes [64]. Rehabilitation programs, particularly cardiac rehabilitation, are highly beneficial for COVID-19 survivors experiencing cardiovascular complications. These programs often include structured exercise training, which is supervised to ensure safety and effectiveness. Exercise improves cardiovascular fitness and alleviates symptoms such as shortness of breath and fatigue [65]. Besides physical training, cardiac rehabilitation programs frequently offer educational components that inform patients about their conditions, treatment options, and necessary lifestyle changes. Psychological support is also integral to rehabilitation, helping patients cope with the emotional and psychological impacts of their experiences during and after COVID-19 [65]. Psychological support is increasingly recognized as vital for the overall well-being of COVID-19 survivors. The pandemic has significantly impacted mental health, which can, in turn, affect cardiovascular health. Interventions, such as cognitive-behavioral therapy (CBT), can help patients address negative thought patterns and behaviors contributing to stress and anxiety [66]. Techniques such as mindfulness, meditation, deep breathing exercises, and yoga effectively reduce stress levels and promote emotional well-being. Support groups also provide valuable opportunities for individuals to connect with others with similar experiences, foster a sense of community, and reduce feelings of isolation [66].

## Conclusions

In conclusion, the long-term cardiovascular impact of COVID-19 is a significant and multifaceted challenge that demands ongoing attention and research. COVID-19's ability to inflict lasting damage on the cardiovascular system through mechanisms such as inflammation, endothelial dysfunction, and direct myocardial injury has been well-documented. These pathophysiological processes result in a variety of clinical manifestations, including myocarditis, heart failure, arrhythmias, and thromboembolic events, which can significantly impair the quality of life and health outcomes of survivors. Comprehensive diagnostic approaches are essential for the early identification and effective management of these conditions. Management strategies must be holistic, incorporating pharmacological and non-pharmacological interventions to address the diverse needs of affected individuals. To tailor interventions appropriately, special consideration must be given to vulnerable populations, including those with pre-existing cardiovascular conditions and different demographic factors. As the pandemic continues to evolve, healthcare providers, researchers, and policymakers must stay informed about the latest developments in the understanding and treatment of long COVID's cardiovascular effects. By fostering a collaborative and multidisciplinary approach, we can improve the long-term health outcomes of COVID-19 survivors and mitigate the ongoing burden of this unprecedented global health crisis.
